# Draft Genome of *Proteus mirabilis* Serogroup O18 Elaborating Phosphocholine-Decorated O Antigen

**DOI:** 10.3389/fcimb.2021.620010

**Published:** 2021-03-25

**Authors:** Grzegorz Czerwonka, Dawid Gmiter, Katarzyna Durlik-Popińska

**Affiliations:** Institute of Biology, Jan Kochanowski University in Kielce, Kielce, Poland

**Keywords:** *Proteus mirabilis*, phosphocholine, lipopolysaccharide, urinary tract infection, genome

## Abstract

*Proteus mirabilis* is a pathogenic, Gram-negative, rod-shaped bacterium that causes ascending urinary tract infections. Swarming motility, urease production, biofilm formation, and the properties of its lipopolysaccharide (LPS) are all factors that contribute to the virulence of this bacterium. Uniquely, members of the O18 serogroup elaborate LPS molecules capped with O antigen polymers built of pentasaccharide repeats; these repeats are modified with a phosphocholine (ChoP) moiety attached to the proximal sugar of each O unit. Decoration of the LPS with ChoP is an important surface modification of many pathogenic and commensal bacteria. The presence of ChoP on the bacterial envelope is correlated with pathogenicity, as decoration with ChoP plays a role in bacterial adhesion to mucosal surfaces, resistance to antimicrobial peptides and sensitivity to complement-mediated killing in several species. The genome of *P. mirabilis* O18 is 3.98 Mb in size, containing 3,762 protein-coding sequences and an overall GC content of 38.7%. Annotation performed using the RAST Annotation Server revealed genes associated with choline phosphorylation, uptake and transfer. Moreover, amino acid sequence alignment of the translated *licC* gene revealed it to be homologous to LicC from *Streptococcus pneumoniae* encoding CTP:phosphocholine cytidylyltransferase. Recognized homologs are located in the O antigen gene clusters of *Proteus* species, near the *wzx* gene encoding the O antigen flippase, which translocates lipid-linked O units across the inner membrane. This study reveals the genes potentially engaged in LPS decoration with ChoP in *P. mirabilis* O18.

## Introduction

*Proteus mirabilis* is an opportunistic Gram-negative bacterial pathogen that swarms across solid surfaces, which often leads to catheter-associated urinary tract infections. The hindered eradication of *P. mirabilis* results in recurrent urinary tract infections (Schaffer and Pearson, 2015). Flagella-based swarming motility and biofilm formation, as well as the production of urease, hemolysin, and lipopolysaccharide (LPS) endotoxin, all contribute to the virulence of *P. mirabilis*. The presence of a long-chain O antigen is important for resistance to normal serum, and is involved in swarming motility as well as the formation of the cell-surface glycocalyx (Knirel et al., 2011). The O antigen consists of repeating polysaccharide units (O units) that typically contain two to eight sugar residues that define the serological specificity of Gram-negative bacteria (Liu et al., 2014). Due to the high structural diversity of *P. mirabilis* O antigens, infections by different serotypes may activate different host immune responses. To date, 83 serogroups within the genus *Proteus* have been identified (Siwińska et al., 2020). An interesting and unique modification of the *P. mirabilis* O antigen is its decoration with phosphocholine (ChoP), which occurs in strains belonging to the O18 serogroup (Fudala et al., 2003). Decoration of glycans with ChoP protects bacteria from innate and adaptive immune system responses, and modifies interactions with host proteins engaged in bacterial pathogenesis (Young et al., 2013).

Biosynthesis of ChoP and decoration of LPS involves four enzymes: choline kinase (LicA), choline transmembrane transporter (LicB), CTP:phosphocholine cytidylyltransferase (LicC) and lipopolysaccharide cholinephosphotransferase (LicD). The first steps in this process is choline uptake from the cell surroundings, involving choline permease LicB and phosphorylation of free choline to ChoP by choline kinase LicA (Young et al., 2013). Choline uptake is carried by both LicB and BetT (a high-affinity choline transporter). The BetT permease contributes to the utilisation of choline in the osmoprotection system, in which choline is oxidized to form glycine betaine that acts as an osmoprotectant, as demonstrated for *Escherichia coli* (Fan et al., 2003). In the LPS decoration pathway, phosphorylated choline is modified *via* the CDP-choline pathway to CDP-ChoP by LicC. This enzyme catalyzes the transfer of a cytidine monophosphate from CTP to phosphocholine to form CDP-choline (Kwak et al., 2002). The final event in the decoration process is CDP-ChoP transfer to a target glycan, mediated by LicD (Young et al., 2013). This enzyme decorates cell surface structures such as LPS in Gram-negative species, e.g. *H. influenzae* and commensal *Neisseria*, the pili of *Neisseria meningitidis*, and lipoteichoic acids and teichoic acids of *S. pneumoniae* (Geiger et al., 2013). Importantly, only in *P. mirabilis* and *Morganella morganii*, the O antigens are decorated with ChoP (Clark and Weiser, 2013).

Our studies revealed the homologs of *licABCD* genes presumably responsible for LPS decoration with ChoP. The biosynthesis of O antigen is regulated by genes which are generally organized in an O antigen gene cluster (Yu et al., 2017). Certain O antigens may contain unique modifications, and genes engaged in their processing are also located within this cluster (Yu et al., 2017). In the Wzx/Wzy-dependent assembly pathway the O antigen repeating units are linked to a lipid carrier (undecaprenyl pyrophosphate) and engage a transmembrane flippase (Wzx) that flips O units from the cytoplasm to the periplasm, where O antigen subunits are polymerized by Wzy. The length of the O antigen chain is regulated by the action of Wzz, a polysaccharide copolymerase (PCP) (Islam and Lam, 2014). The conservation of the O antigen gene cluster among bacteria is low, and heterogeneity of this region results in the synthesis of different O antigens (Islam and Lam, 2014). The low conservation between *wzy* genes of different serotypes enables the use of this gene as a molecular marker for molecular serotyping (Wang et al., 2017).

The first complete genome of *P. mirabilis* was characterised for the widely-studied strain HI4320 (serogroup O28) isolated from the urine of a patient with a long-term indwelling urinary catheter. Genome annotation revealed the coding sequences and genomic locations of previously-characterized virulence determinants. From this annotation, genes predicted to be involved in LPS biosynthesis were identified (Pearson et al., 2008). Subsequent studies have reported that *Proteus* serogroups can be genetically distinguished based on the sequences of their respective O antigen biosynthesis clusters; however, information about the O18 serogroup was incomplete (Yu et al., 2017). Here we report the draft genome sequence of *P. mirabilis* strain PrK 34/57 belonging to the O18 serogroup, including the fully-sequenced and annotated O antigen biosynthesis gene cluster.

## Materials and Methods

### Bacterial Strains and DNA Extraction

*P. mirabilis* strain PrK 34/57 (O18) was obtained from the Czech National Collection of Type Cultures in Prague, Czech Republic. Cells were grown overnight in 50 ml of LB (Biocorp, Warsaw, Poland), diluted in ratio 1:100 with fresh LB medium and cultivated for following tests at 37°C with shaking (160 rpm) for 12–18 h in an Ecotron incubator (Infors HT, Basel, Switzerland). Genomic DNA was isolated with a GenElute™ Bacterial Genomic DNA Kit (Sigma-Aldrich, Saint Louis, MO, USA) according to manufacturer’s protocol from 1.5 ml of overnight culture. Final elution was performed with 100 μl of nuclease-free water. DNA quality was assessed using a NanoDrop 2000 Spectrophotometer (Thermo Fisher Scientific, Waltham, MA, USA).

### Sequencing

Libraries were prepared using the Nextera XT kit (Illumina Inc., San Diego, CA, USA) according to the manufacturer’s protocol. Libraries were sequenced on the NextSeq machine (Illumina) with 2 × 150 bp paired-end reads with a depth of 200-fold coverage. Over 91.00% of bases of sequencing reads had quality scores ≥Q30.

### Genome ‘*De Novo*’ Assembly

The trimmed Illumina reads were assembled using Unicycler, an assembly pipeline for bacterial genomes (Wick et al., 2017). The Unicycler Version 0.4.8.0 was available online on the Galaxy web Server (https://usegalaxy.org/). The Unicycler pipeline functions as a SPAdes-optimizer. For the assembly, the default options of SPADes were selected, that includes turned on error correction, the *k*-mer in a range of 0.2 to 0.95 (expressed as a fraction of the read length). Contigs with a fraction of the chromosomal depth lower than 0.25 were filtered out. In terms of Unicycler options, the Normal Bridge mode (moderate contigs size and moderate misassembly rate) was selected. Contigs shorter than 500 bp were excluded from the final assembly.

### Genome Annotation

The obtained contigs were reordered relative to the *P. mirabilis* HI4320 reference genome using Mauve Contig Mover (MCM) of Mauve 2.4.0 software to facilitate the study (Darling et al., 2004; Rissman et al., 2009). The default parameters were used. Genome sequence of PrK 34/57 was primarily functionally annotated by Rapid Annotation Subsystems Technology (RAST) server using the ClassicRAST annotation scheme, FIGfams version 70, automatic error correction, and automatic frame shift correction (Aziz et al., 2008; Gmiter et al., 2019). Further, during WGS (Whole Genome Shotgun) submission to NCBI, it was annotated using the NCBI Prokaryotic Genome Annotation Pipeline (PGAP) (Tatusova et al., 2016) to ensure better insight into the genomic features.

### Variant Calling Analysis

For variant calling analysis of PrK 34/57, the Illumina reads were tested using Snippy (Galaxy Version 4.4.5+galaxy2) with the default parameters and HI4320 as a reference genome (https://github.com/tseemann/snippy) (Bush et al., 2020).

### Phylogenetic Analysis

In order to perform the *P. mirabilis* PrK 34/57 comparative study, selected *P. mirabilis*, both complete and WGS type, genomes were downloaded from NCBI and presented in **Table 1**. For WGS genomes, the reordering of contigs was performed using MCM as above. The comparative genomics include the phylogenomic analysis based on average nucleotide identities (ANI) using FastANI algorithm (Jain et al., 2018) and single nucleotide polymorphisms (SNPs) using CSI Phylogeny webserver (Kaas et al., 2014). The CSI Phylogeny webserver was used with the default options, including minimum depth at SNP positions = 10×, minimum relative depth at SNP positions = 10%, minimum distance between SNPs = 10 bp, minimum SNP quality = 30, minimum read mapping quality = 25 and minimum Z-score = 1.96. The obtained phylogeny tree includes the *P. mirabilis* HI4320 as a reference genome. The tree resulted from the CSI Phylogeny webserver was visualized using Interactive Tree Of Life (iTOL) version 5 (Letunic and Bork, 2019). To determine global rearrangement structure between *P. mirabilis* PrK 34/57 and other genomes, the progressiveMauve option of Mauve was used (Darling et al., 2004; Darling et al., 2010). The backbone file, an output of Mauve, was used to visualize the genomes comparison with the R package genoPlotR (Guy et al., 2011).

**Table 1:** *Proteus mirabilis* genomes used in the study.

Strain	Accession number	Genome size (bp)	Reference
HI4320	AM942759	4,063,606	Pearson et al. (2008)
BB2000	CP004022	3,846,754	Sullivan et al. (2013)
GN2	CP026581	4,012,640	Li et al. (2018)
BC11-24	CP026571	4,021,165	Lei et al. (2018)
K1609	CP028522	3,817,795	Gmiter et al. (2019)
K670	CP028356	3,935,626	Gmiter et al. (2019)
Pr2921	LGTA00000000	3,924,499	Giorello et al. (2016)
1230_SSON	NZ_JVXV01000000	3,923,692	Roach et al. (2015)
PM_125	NZ_LWUL00000000	3,955,474	Yu et al. (2016)

### KEGG Annotation

Analysis of KEGG pathways in PrK 34/57 was conducted by GhostKOALA, an automated metagenome annotation server that characterizes gene functions and pathways based on KEGG Orthology sequence assignments (Kanehisa et al., 2016). As an input file, the Amino-Acid FASTA file generated by RAST was used.

### The Virulome Investigation

The presence of virulence driving genes was investigated using a local data base created with the makeblastdb option of BLAST+ (Camacho et al., 2009). Genes for data base construction were selected based on previously-described genome of *P. mirabilis* HI4320 strain. The database included genes responsible for ureolitic, proteolitic and hemolytic activity, motility (flagellum synthesis and chemotaxis), and fimbriae synthesis.

### Resistance Gene Identifier

The resistome of *P. mirabilis* PrK 34/57 was predicted from nucleotide data based on homology and SNP models using the Resistance Gene Identifier (RGI) based on The Comprehensive Antibiotic Resistance Database (CARD; https://card.mcmaster.ca) (Alcock et al., 2020). The DNA was used as a data type, selection criteria was selected as perfect and strict hits only, the nudges above 95% was excluded, and the sequence quality was selected as a high coverage.

### Genome Accession Number

This Whole Genome Shotgun Project of *P. mirabilis* PrK 34/57 has been deposited at GenBank (http://www.ncbi.nlm.nih.gov) under the accession JAAMPE000000000. The version described in this paper is version JAAMPE010000000.

## Results

### Genome Overview

**Figure 1:**
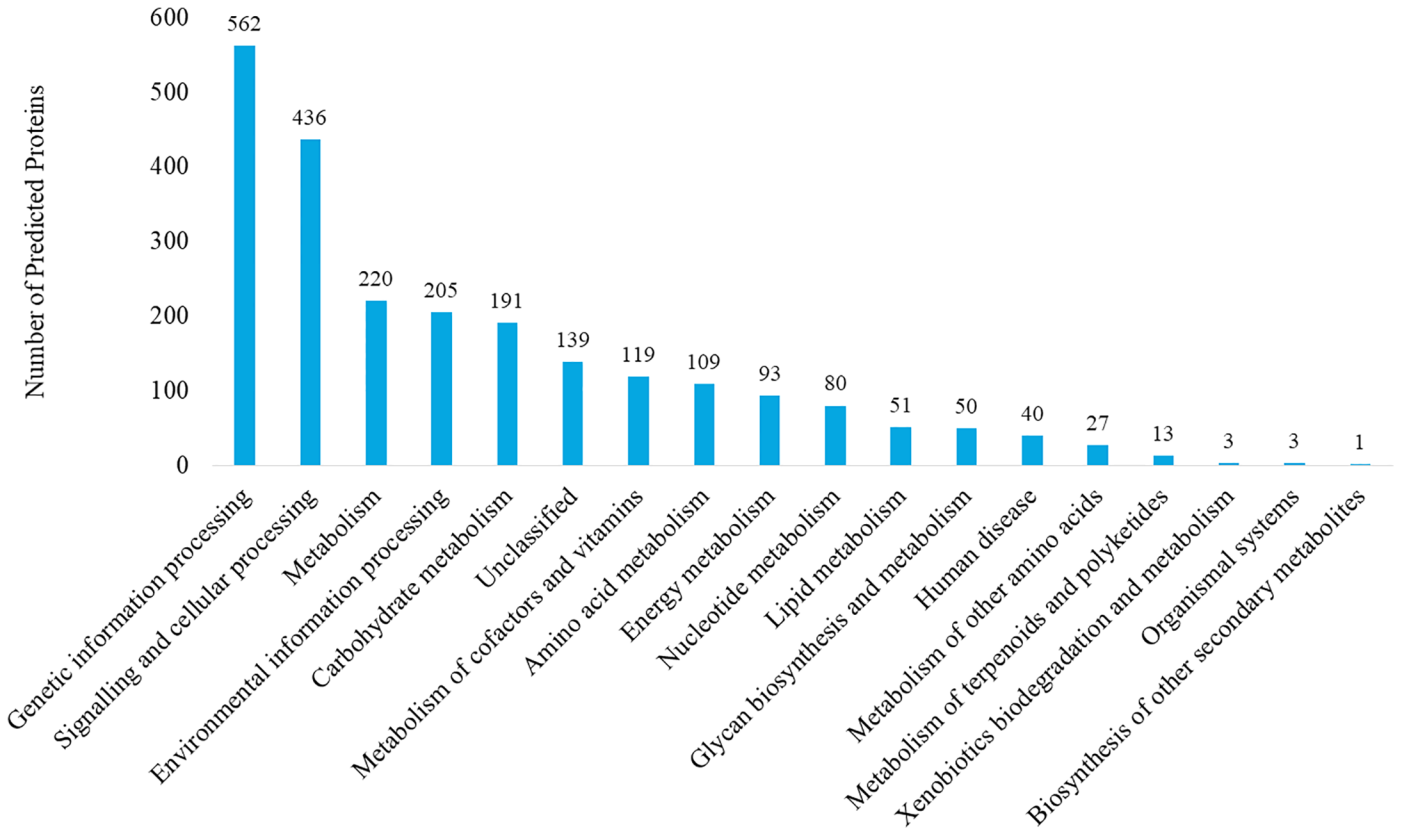
KEGG pathway analysis of *Proteus mirabilis* PrK 34/57 strain. Proteins were identified and categorized using the GhostKOALA tool against the Amino-Acid file generated by RAST server.

The genome of *P. mirabilis* strain PrK 34/57 was determined to be 3,970,593 bp in length, with a GC content of 38.7%. It was assembled into 60 contigs (>500 bp). Based on PGAP annotation, there are 3,722 genes, of which 3,594 (96.6%) are protein-coding genes, 56 (1.5%) are pseudogenes and the remaining 72 are predicted RNA-coding genes, including 66 tRNAs, 2 rRNAs and 4 ncRNAs. The general features of *P. mirabilis* PrK 34/57 assembly identified by RAST are presented in **Table 2**. In contrast to PGAP, 3,620 genes were annotated using RAST, which were assigned to 497 subsystems. Importantly, among virulence, disease and defence subsystem categories, we identified genes involved in bacteriocin tolerance and production, as well as tolerance to antibiotics and heavy metals (including efflux pumps). Moreover, PrK 34/57 possesses 56 genes related to iron acquisition and metabolism. Additionally, there is a set of 57 genes related to motility and chemotaxis, both of which are important for the ability of *P. mirabilis* strains to swarm over solid surfaces. We also identified genes encoding urease subunits (nitrogen metabolism category), an important enzyme for *P. mirabilis* survival in the urinary tract. Furthermore, genes involved in choline transport and metabolism were identified (miscellaneous category). The detailed subsystems present in PrK 34/57 are summarised in **Table 3**. Variant calling is a useful comparative genomic method that provides insight into organismal differences at the nucleotide level (Fariq et al., 2019). A set of variants are identified from tested and reference genomes, including small polymorphisms, specifically single-nucleotide polymorphisms (SNPs), insertions and deletions (indels), multi-nucleotide polymorphisms (MNPs) and complex events (composite insertion and substitution events) smaller than the length of short-read sequencing alignment. During our current analysis, 18,240 variants were identified between PrK 34/57 and HI4320. For all variants, their FILTERs status was PASS, indicating that variants in the raw data are true calls and not false positives resulting from low coverage depth. Observed variants were divided into five categories, and numbers and frequencies are summarized in **Table 4**. Using GhostKOALA annotation server totally 2342 entries (64.7%) was annotated in the genome of PrK 34/57. The distribution and frequency of KEGG pathways was presented on **Figure 1**.

**Table 2:** *Proteus mirabilis* PrK 34/57 genome assembly statistics based on RAST annotation server.

Attribute	Value
Genome size	3,970,593
%GC	38.7
N50 (bp)	203,813
L50 (bp)	8
Number of contigs (with PEGs)	60
Number of subsystems	497
Number of coding sequences	3,620
Number of RNAs	70

**Table 3:** Subsystem distribution of *Proteus mirabilis* PrK 34/57 via RAST server-based annotation.

Subsystems	Counts
Cofactors, Vitamins, Prosthetic groups, Pigments	249
Cell wall and Capsule	161
Virulence, Disease and Defense	73
Potassium metabolism	25
Photosynthesis	0
Miscellaneous	45
Phages, Prophages, Transposable elements, Plasmids	62
Membrane transport	173
Iron acquisition and metabolism	56
RNA metabolism	223
Nucleosides and Nucleotides	100
Protein metabolism	273
Cell division and Cell cycle	38
Motility and Chemotaxis	57
Regulation and Cell signalling	99
Secondary metabolism	4
DNA metabolism	105
Fatty acids, Lipids, and Isoprenoids	110
Nitrogen metabolism	26
Dormancy and Sporulation	6
Respiration	151
Stress response	132
Metabolism of Aromatic compounds	3
Amino acids and Derivatives	367
Sulfur metabolism	37
Sulfur metabolism	35
Carbohydrates	327

**Table 4:** Summary of variants in *Proteus mirabilis* PrK 34/57 genome against *P. mirabilis* HI4320 reference strain.

	Total number of variations	Categories				
		SNPs	MNPs	Insertions	Deletions	Complex
Count	18,240	16,489	245	106	117	1283
Frequency (%)	100	90.40	1.34	0.58	0.64	7.03

### Phylogenetic Analysis

The fastANI analysis of the *P. mirabilis* PrK 34/57 genome revealed high similarity with other genomes. ANI values were 98.6077 to 99.1747% compared with BC11-24 and K670 genomes, respectively. These values far exceed the generally accepted 95% cut-off level for taxonomy affiliation of newly sequenced genomes (Figueras et al., 2014). The results of detailed comparison of the genomes are presented in **Table 5**.

**Table 5:** ANI similarity matrix between studied *Proteus mirabilis* genomes.

	1230_SSON	BB2000	BC11-24	GN2	HI4320	K670	K1609	PM_125		Pr2921	PrK 34/57
1230_SSON	100	98.7973	98.6261	98.8935	99.1878	99.0408	98.7498		99.0670	98.9698	99.0348
BB2000	98.7973	100	99.0143	98.8578	98.8301	98.8168	99.3476	98.8259		98.7436	98.7991
BC11-24	98.6261	99.0143	100	98.6209	98.6625	98.6047	98.8936	98.5968		98.5448	98.6077
GN2	98.8935	98.8578	98.6209	100	98.8563	99.0234	98.7216	98.8451		98.9670	98.9807
HI4320	99.1878	98.8301	98.6625	98.8563	100	98.9659	98.7721	99.0081		98.9928	98.9781
K670	99.0408	98.8168	98.6047	99.0234	98.9659	100	98.7839	98.8571		99.0467	99.1747
K1609	98.7498	99.3476	98.8936	98.7216	98.7721	98.7839	100	98.6677		98.7019	98.7622
PM_125	99.0670	98.8259	98.5968	98.8451	99.0081	98.8571	98.6677	100		98.8820	98.9628
Pr2921	98.9698	98.7436	98.5448	98.9670	98.9928	99.0467	98.7019	98.8820		100	99.0904
**PrK_34_57**	**99.0348**	**98.7991**	**98.6077**	**98.9807**	**98.9781**	**99.1747**	**98.7622**	**98.9628**		**99.0904**	**100**

Values represent the average nucleotide identity in percent. The ANI values for *P. mirabilis* PrK 34/57 strain are indicated by bold font.

**Figure 2:**
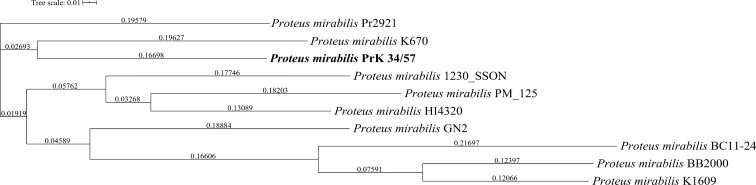
Whole genome phylogeny of *Proteus mirabilis* strains based on single nucleotide polymorphisms (SNPs) using CSI Phylogeny webserver. The sequence of studied *P. mirabilis* PrK 34/57 strain is indicated by bold font. The obtained Newick file was visualized using Interactive Tree Of Life (iTOL) version 5.

**Figure 3:**
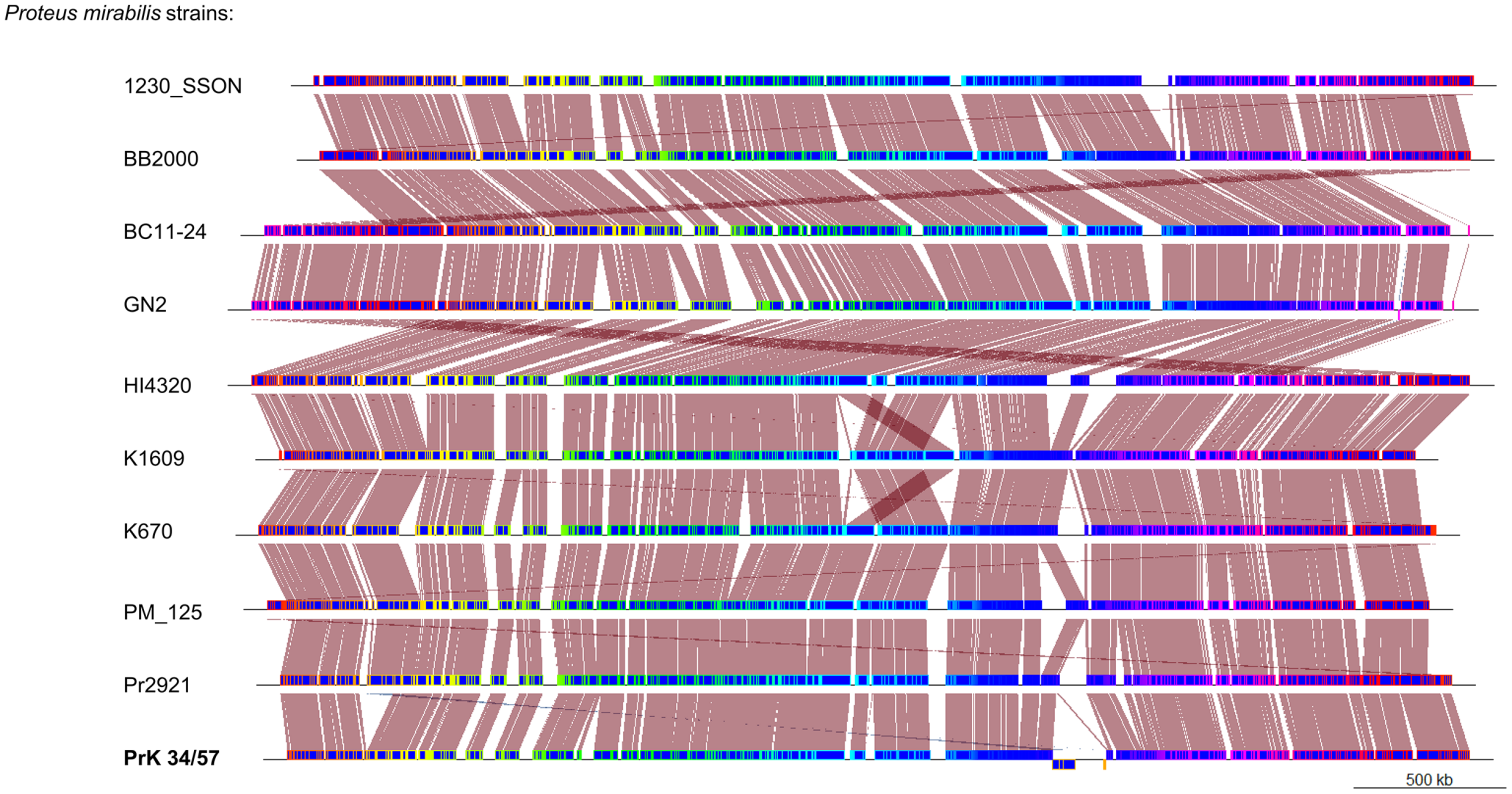
Whole genome comparison of *Proteus mirabilis* genomes using progressiveMauve option of Mauve software. Genomes are represented in blue, and blocks with borders of different colors are homologous between genomes The backbone file was visualized using the R package genoPlotR.

A phylogenetic tree based on SNPs identified from whole genome comparisons (**Figure 2**) resulted in three major clades: PrK 34/57; the HI4320 reference genome; and BB2000, another frequently studied *P. mirabilis* strain (Sullivan et al., 2013). The PrK 34/57 genome is most similar to the previously reported genome of K670 (Gmiter et al., 2019), and shares greater similarity with HI43200 than BB2000. These observations correspond to the results of ANI-based analysis. Similar results were obtained following analysis of whole genome phylogeny of *P. mirabilis* strains based on the Mauve comparison (data not shown). The global rearrangement structure of the studied *P. mirabilis* genomes is visualized in **Figure 3**. Significant similarity between genomes is evident, with only a few genome rearrangement events. These occasional events are illustrated by the crossing lines that link Locally Collinear Blocks (LCBs). LCBs were calculated by Mauve to identify conserved segments that appear to be internally free from genome rearrangements. However, the PrK 34/57 genome displays blocks below the genome’s center line, which suggests the presence of inverted regions in PrK 34/57 (regions that align in the reverse complement (inverse) orientation) compared with other strains (Darling et al., 2004).

### The Virulome Investigation

To better explore virulence potential of *P. mirabilis* strain PrK 34/57 the presence of genes involved in expression of most important virulence factors was tested using local virulence factor databases. From this analysis, genes associated with all major virulence factors were detected in PrK 34/57. The sequence similarity with HI4320 was up to 100% depending on gene class. Interestingly, though previously detected in strain HI4320, genes encoding fimbriae 3 (*fim3A*) were not detected in PrK 34/57 (**Table 6**).

**Table 6:** Presence of genes related to *Proteus mirabilis* virulence in PrK 34/57.

Virulence factor		Presence/absence
Enzymatic activity	Ureolytic activity	**+**
	Proteolytic activity	**+**
	Haemolytic activity	**+**
Motility and chemotaxis	Flagellum	**+**
	Chemotaxis	**+**
Fimbriae and pili:	MR/P Fimbriae	**+**
	mrp'	**+**
	Fimbriae 3	**-**
	UCA (NAF)	**+**
	Fimbriae 5	**+**
	Fimbriae 6	**+**
	Fimbriae 7	**+**
	Fimbriae 8	**+**
	PMF(MR/K)	**+**
	Fimbriae 10	**+**
	PMP	**+**
	Fimbriae 12	**+**
	ATF	**+**
	Fimbriae 14	**+**
	Fimbriae 15	**+**
	Fimbriae 16	**+**
	Fimbriae 17	**+**

### Identification of Putative Antibiotic Resistance Genes

Based on the Comprehensive Antibiotic Research Database (CARD), the Resistance Gene Identifier (RGI) tool identified homologs of genes responsible for resistance to aminoglycosides (amikacin, tobramycin) and beta-lactams (ampicillin, piperacillin, amoxicillin), and the results are presented in **Table 7**. Antimicrobial peptides resistance in this strain is presumably supported by homologs of *Klebsiella pneumoniae* KpnFH efflux pumps (Poirel et al., 2017; Aghapour et al., 2019).

**Table 7:** The putative antibiotic resistance genes identified in genome of *P. mirabilis* PrK 34/57 by the Resistance Gene Identifier (RGI) based on the Comprehensive Antibiotic Research Database (CARD).

ARO Term*	SNP	Detection Criteria	AMR Gene Family	Drug Class	Resistance Mechanism	% Identity of Matching Region	% Length of Reference Sequence
*adeF*		protein homolog model	resistance-nodulation-cell division (RND) antibiotic efflux pump	fluoroquinolone antibiotic, tetracycline antibiotic	antibiotic efflux	42.17	99.34
*tet(D)*		protein homolog model	major facilitator superfamily (MFS) antibiotic efflux pump	tetracycline antibiotic	antibiotic efflux	53.79	101.02
CRP		protein homolog model	resistance-nodulation-cell division (RND) antibiotic efflux pump	macrolide antibiotic, fluoroquinolone antibiotic, penam	antibiotic efflux	98.1	100.00
*catA4*		protein homolog model	chloramphenicol acetyltransferase (CAT)	phenicol antibiotic	antibiotic inactivation	96.77	100.00
*rsmA*		protein homolog model	resistance-nodulation-cell division (RND) antibiotic efflux pump	fluoroquinolone antibiotic, diaminopyrimidine antibiotic, phenicol antibiotic	antibiotic efflux	92.98	101.64
*Klebsiella pneumoniae KpnH*		protein homolog model	major facilitator superfamily (MFS) antibiotic efflux pump	macrolide antibiotic, fluoroquinolone antibiotic, aminoglycoside antibiotic, carbapenem, cephalosporin, penam, peptide antibiotic, penem	antibiotic efflux	72.71	100.00
*Klebsiella pneumoniae KpnF*		protein homolog model	major facilitator superfamily (MFS) antibiotic efflux pump	macrolide antibiotic, aminoglycoside antibiotic, cephalosporin, tetracycline antibiotic, peptide antibiotic, rifamycin antibiotic	antibiotic efflux	68.81	100.92
*Haemophilus influenzae* PBP3 conferring resistance to beta-lactam antibiotics	D350N	protein variant model	Penicillin-binding protein mutations conferring resistance to beta-lactam antibiotics	cephalosporin, cephamycin, penam	antibiotic target alteration	51.45	98.03
*Morganella morganii gyrB* conferring resistance to fluoroquinolones	S463A	protein variant model	fluoroquinolone resistant gyrB	fluoroquinolone antibiotic	antibiotic target alteration	84.58	100.00
*Escherichia coli* EF-Tu mutants conferring resistance to Pulvomycin	R234F	protein variant model	elfamycin resistant EF-Tu	elfamycin antibiotic	antibiotic target alteration	94.91	96.09

^*^ARO, Antibiotic Resistance Ontology.

### Organization of the *P. mirabilis* O18 Antigen Gene Cluster

**Figure 4:**
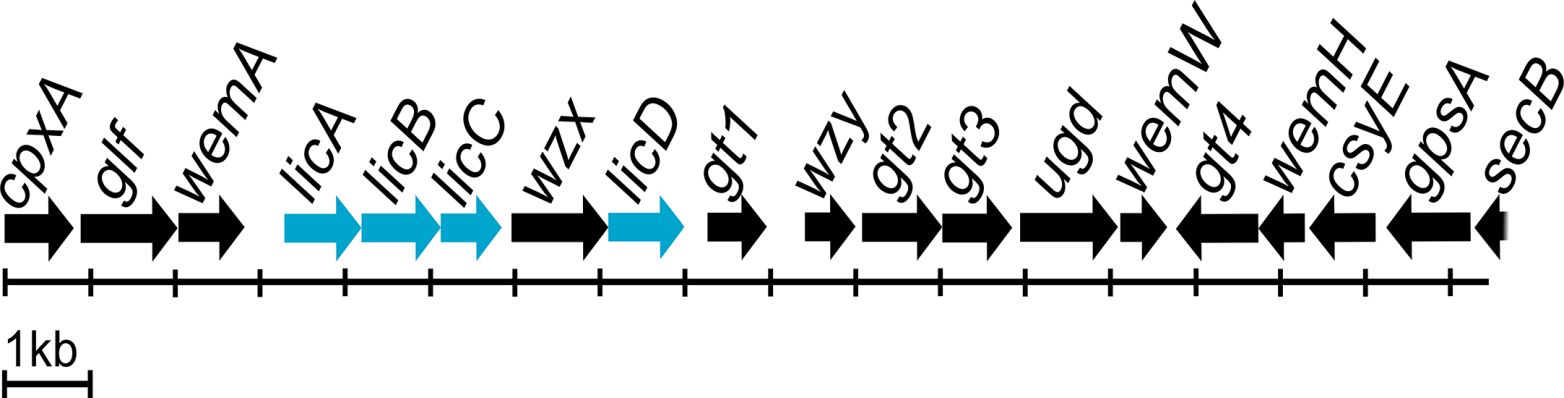
*Proteus mirabilis* O18 O antigen gene cluster arrangement. Highlighted genes represent *licABCD* genes and its organisation in the Wzx/Wzy-dependent O antigen biosynthesis gene cluster. Scheme modified from Yu et al. (2017).

Most O antigen gene clusters are located at fixed positions on bacterial chromosomes, and genetic variation in these gene clusters is largely responsible for the diversity of O antigen forms (Liu et al., 2008). There are three types of molecular biosynthesis pathways for O antigens: Wzx/Wzy-dependent, synthase-dependent, and ABC transporter-dependent pathways (Bertani and Ruiz, 2018). The Wzx/Wzy-dependent pathway is the most common, and the main components are the integral inner membrane flippase Wzx, the polymerase Wzy, and the chain-length regulator protein Wzz (Islam and Lam, 2014; Yu et al., 2017). The *P. mirabilis* O antigen gene cluster is located between *cpxA* and *secB* genes, and contains both the *wzx* and *wzy* genes (Wang et al., 2010; Yu et al., 2017). Genome annotation using the RAST server identified the genes involved in choline transport and metabolism. Three of the genes were *licA* located at open reading frame (ORF) ORF04, *licB* located at ORF05, and *licD* located at ORF08 based on the cluster scheme proposed by Yu and colleagues (Yu et al., 2017). The O antigen cluster contained one additional ORF (ORF06); amino acid sequence analysis by Phyre2 software (Kelley et al., 2015) revealed 98% coverage, and a 224 amino acid polypeptide was modelled with 100.0% confidence with ctp:phosphocholine cytidylytransferase (LicC) as the single highest scoring template. The *licABC* genes overlap with each other, suggesting they are co-transcribed during the O antigen assembly process. The sequence of the *licD* gene overlaps with that of the *wzx* gene. Both regions are divided by a short intergenic sequence. The *licABC* genes are localized between the *wemA* gene, encoding a UDP-galactofuranosyl transferase according to Phyre2 (confidence 98.1%, coverage 49%), separated from *licD* by *wzx* encoding a flippase. The complete O antigen biosynthesis cluster for the *P. mirabilis* O18 serogroup is presented on **Figure 4**.

## Discussion

*P. mirabilis* is a Gram-negative, commensal bacterium, which is frequently responsible for catheter-associated urinary tract infections. In this work, we determined the draft genome of *P. mirabilis* strain PrK 34/57 (O18) containing the unique ChoP modification of O antigen. Furthermore, the obtained data allowed us to investigate the general features of the studied genome. PGAP and RAST annotation revealed the presence of important genes involved in *P. mirabilis* pathogenicity, including genes responsible for swarming motility, ureolytic activity and iron acquisition (Różalski et al., 1997). The structure and organisation of the PrK 34/57 genome was compared with other *P. mirabilis* strains, and the genome of PrK 34/57 is generally similar in terms of features such as genome size, GC ratio and the number of coding sequences (Pearson et al., 2008; Sullivan et al., 2013; Shi et al., 2016; Gmiter et al., 2019). In the present study, the HI4320 genome was used as a reference, since this strain has been studied extensively (Armbruster et al., 2018). Other complete and WGS genomes were also included to better understand the general position of PrK 34/57 among *P. mirabilis* isolates from around the world. *P. mirabilis* PrK 34/57 shares high similarity with strain HI4320, but it is most closely related to the previously studied K670 strain. Simple variant calling analysis was performed using HI4320 and PrK 34/57. The results indicated few *P. mirabilis* PrK 34/57 polymorphisms (<20,000 variants), especially considering the high level of polymorphisms revealed in previously studied *Acidithiobacillus ferrooxidans* strains (>70,000 variants) (Fariq et al., 2019). These variant calling analyses may be relevant for future studies on PrK 34/57. Similar conclusions about the high genetic relatedness between PrK 34/57 and other strains can be drawn from the ANI results, which were in agreement with previous observations (Armbruster et al., 2018).

The obtained draft genome sequence of ChoP-containing *P. mirabilis* O18 strain PrK 34/57 is important in a serological context and will be beneficial during subsequent studies of the pathogenicity of this bacterium. Choline uptake and LPS decoration protects bacteria from osmotic pressure, and enables avoiding the immune system recognition. Choline uptake is provided in *P. mirabilis* O18 by a high-affinity choline transport protein BetT. Choline gained in this way is involved in the glycine betaine biosynthesis pathway, which acts as an osmoprotectant (Deole and Hoff, 2020). The other way for choline recruitment into the cell is the ChoP decoration pathway, where *licABC* genes products (kinase, permease, and cytidylyltransferase, respectively) incorporate, and modify choline molecule. In the next step, CDP-choline is attached to the target glycans by the transferase LicD (Young et al., 2013). In *P. mirabilis* O18 antigen gene cluster genes *licABC* overlaps, which suggest that they are organised in a separate cluster, and expressed simultaneously. The action of this cluster may result in incorporation choline into the cell and its modification to CDP-choline. The *licD* encoding transferase overlaps with the sequence of the *wzx* flippase, which suggests that both genes are co-transcribed, and expression of *licD* depends on expression of *wzx* and is regulated with the same factors. This finding reveals that ChoP attachment to O antigen repeating unit in *P. mirabilis* is probably regulated by two independent regulatory regions, upstream the *licABC* and *wzx/licD* clusters, and it occurs as two independently controlled events. The decoration of a single O antigen unit presumably occurs before flipping of the undecaprenyl phosphate-linked O units to the periplasmic face of the inner membrane by the Wzx flippase.

In conclusion, determination of the *P. mirabilis* O18 genome sequence revealed the presence and localisation of genes *licABCD* potentially involved in choline uptake, modification and decoration of LPS. Enzymes involved in ChoP decoration, a choline kinase, choline permease, and ctp:phosphocholine cytidylytransferase are encoded by the *licABC* cluster. This cluster is separated from *licD* homolog (encoding lipopolysaccharide cholinephosphotransferase) by the *wzx* gene encoding a crucial LPS biosynthesis transmembrane flippase. The results allowed us to present the provisional organisation of the O antigen gene cluster in the *P. mirabilis* O18 serogroup.

## Data Availability Statement

The datasets presented in this study can be found in online repositories. The names of the repository/repositories and accession number(s) can be found in the article/supplementary material.

## Author Contributions

GC—carried out the experiments with DNA isolation, and genome analysis in order to investigate the presence of *licABCD* gene and organisation the O antigen gene cluster as well as antibiotic resistance identification. Contributed to the interpretation of the results, and took the lead in writing the manuscript. DG—carried out DNA isolation, genome annotation and deposition in GenBank. Carried out the bioinformatic analysis of general features of reported genome, interpretation of the results, and writing the manuscript. KD-P—Contributed to the interpretation of the results, and contributed in writing the manuscript. All authors provided critical feedback and helped shape the research, analysis, and manuscript. All authors contributed to the article and approved the submitted version.

## Funding

This research was supported by the Polish National Science Centre (Grant No. 2017/01/X/NZ6/01141), and Jan Kochanowski University Rector’s Grant 2019 (no SIGR.RN.20.061.604). DG received the PhD scholarship from the NSC Grant No. 2019/32/T/NZ1/00515.

## Conflict of Interest

The authors declare that the research was conducted in the absence of any commercial or financial relationships that could be construed as a potential conflict of interest.
